# QTL dissection and mining of candidate genes for *Ascochyta fabae* and *Orobanche crenata* resistance in faba bean (*Vicia faba* L.)

**DOI:** 10.1186/s12870-021-03335-5

**Published:** 2021-11-22

**Authors:** Natalia Gutierrez, Ana M. Torres

**Affiliations:** Área de Genómica y Biotecnología, IFAPA-Centro Alameda del Obispo, Apdo 3092, E-14080 Córdoba, Spain

**Keywords:** Faba bean, Ascochyta, *Orobanche*, Resistance, Map saturation, Medicago, Sinteny, QTL interval, Fine-mapping, Candidate genes

## Abstract

**Background:**

Ascochyta blight caused by *Ascochyta fabae* Speg. and broomrape (*Orobanche crenata)* are among the economically most significant pathogens of faba bean. Several QTLs conferring resistance against the two pathogens have been identified and validated in different genetic backgrounds. The aim of this study was to saturate the most stable QTLs for ascochyta and broomrape resistance in two Recombinant Inbred Line (RIL) populations, 29H x Vf136 and Vf6 x Vf136, to identify candidate genes conferring resistance against these two pathogens.

**Results:**

We exploited the synteny between faba bean and the model species *Medicago truncatula* by selecting a set of 219 genes encoding putative WRKY transcription factors and defense related proteins falling within the target QTL intervals, for genotyping and marker saturation in the two RIL populations. Seventy and 50 of the candidate genes could be mapped in 29H x Vf136 and Vf6 x Vf136, respectively. Besides the strong reduction of the QTL intervals, the mapping process allowed replacing previous dominant and pedigree-specific RAPD flanking markers with robust and transferrable SNP markers, revealing promising candidates for resistance against the two pathogens.

**Conclusions:**

Although further efforts in association mapping and expression studies will be required to corroborate the candidate genes for resistance, the fine-mapping approach proposed here increases the genetic resolution of relevant QTL regions and paves the way for an efficient deployment of useful alleles for faba bean ascochyta and broomrape resistance through marker-assisted breeding.

**Supplementary Information:**

The online version contains supplementary material available at 10.1186/s12870-021-03335-5.

## Background

Faba bean (Vicia faba L.) is one of the most important legume crops known for its high-yield potential and seed protein content. Also referred to as horse bean or broad bean, faba bean is a common staple food in Mediterranean countries, India, Pakistan and China. Faba bean is today the fifth most widely grown cool season legume [1] and is well suited for sustainable farming practices in temperate or cool environments. One of the key environmental benefits of faba bean is the capacity to establish symbiosis with Rhizobium bacteria resulting in biological nitrogen fixation that improves soil fertility and reduces the input of fertilizers. Faba bean cultivation in intensive cereal-dominated crop rotations further provides a break of disease cycles and allows efficient control of weeds. Moreover, the crop supplies multiple ecological and environmental services by hosting associated organisms such as wild insects and soil microbes that can positively affect the sustainability of agroecosystems.

Despite the ability of this species to adapt to diverse climates, faba bean yield remains unstable due to susceptibility to pests and diseases that reduce crop production and affect the commercialization of the seeds. Ascochyta blight is a major foliar necrotrophic fungal disease, and together with the parasitic weed *Orobanche* spp. ranks among the main constraints for faba bean production.

Ascochyta blight is widespread and of economic concern in mild and wet weather conditions. The causal agent *Ascochyta fabae* Speg. (teleomorph *Didymella fabae* Jellis and Punithalingam) produces lesions on leaves, stems and pods lesions which may also spread to the seed. Foliar infections can lead to yield losses ranging between 30 and 90%, which can be further aggravated by seed staining and mold resulting in a failure to meet requirements in export markets [2–5]. Although ascochyta blight can be controlled by fungicides, the economically most viable and environmentally sustainable method of control is the development of disease resistant varieties. On the other hand, broomrape (*Orobanche crenata*) represents the major constraint for faba bean cultivation in the Mediterranean basin and Middle East. This root holoparasite attaches to the roots of the host plant to take water and nutrients, causing losses in crop yield that can reach up to 100% depending on infection severity and the sowing date [6]. The only effective way to control parasitic weeds is an integrated approach including cultural practices such as delayed sowing, hand weeding, no-tillage, nitrogen fertilization, intercropping or rotations [7]. However, the major control methods currently available to farmers are the use of resistant varieties and chemical control, although both have their limitations.

Disease resistances are difficult traits in terms of genetics and breeding due to the often polygenic inheritance and the complexity of the disease evaluation. Previous studies identified and validated stable quantitative trait loci (QTLs) for *A. fabae* and *Orobanche* resistance in different faba bean genetic backgrounds and populations [8–13]. However, the resolution of the genetic maps remains low, thus limiting the accuracy of QTL mapping and the application of marker assisted selection (MAS) approaches.

Fine mapping and discovery of candidate genes underlying ascochyta and broomrape resistance are important for modern faba bean breeding, but the enormous size (13 Gb) and repetitive nature of the faba bean genome hampers sequence assembly/annotation and the development of high-density maps, making it very difficult to pinpoint loci responsible for specific traits. Recently, innovative strategies such as synteny analysis, translational genomics or transcriptome sequencing have been used for fine mapping of key QTL regions. For example, transcriptome profiling during ascochyta infection of faba bean provided gene-based SNPs that were applied towards targeted genomic studies on disease resistance/tolerance [14, 15]. Using the SuperSAGE technique, jasmonic acid (JA) signaling and pectin esterase-encoding genes were identified [14]. A subsequent *de novo* transcriptome assembly identified transcripts, such as LRR proteins and Rho2 GTPase-activating protein 2 (RGA2), that were significantly linked to susceptible and resistant interactions between faba bean and *A. fabae* [15]. In a more recent study [16], 92 SNPs derived from faba bean transcripts differentially expressed during ascochyta infection were mapped together with a set of pathogenesis related proteins in the recombinant inbred line (RIL) population of a cross between the lines 29H and Vf136. The new SNP markers map within or nearby the most stable QTLs intervals in chromosomes (chrs.) II, III and VI, allowing to replace prior pedigree-specific, dominantly-inherited RAPD flanking markers with more robust and transferrable gene-based markers. This study also uncovered genomic regions encoding known resistance mechanisms, providing targets for selection strategies against these two pathogens. Nevertheless, the confidence intervals of the QTLs are still large, ranging from 12 to nearly 50 cM. To increase the saturation of these target regions, a more accurate dissection of the disease QTL intervals is needed to reduce the number of positional candidate genes for further functional analysis.

Numerous studies have identified WRKY transcription factors (TFs) as key players in plant resistance of particular interest, which are involved in diverse biotic/abiotic stress responses [17–19]. WRKYs also play important roles in modulating the balance and crosstalk between distinct plant defense pathways regulated by salicylic acid (SA), jasmonic acid (JA) and ethylene (ET), which trigger immune responses to different pathogens [20]. Several WRKY genes in chickpea (*Cicer arietinum* L.) and barrel medic (*Medicago truncatula*) were identified and observed differential regulation of many chickpea group-III WRKY genes during *Ascochyta rabiei* infection and SA treatment [18]. Similarly, several defense-related genes were suggested to be involved in sunflower defense against broomrape [21]. Because of their tight regulation involved in specific recognition and binding to downstream promoters, WRKY TFs are promising candidates for crop improvement [19] and could be utilized in improvement of faba bean disease resistance, as previously reported in soybean and chickpea [18, 22].

In this study we exploited the synteny between faba bean and model or related legume species to increase the genetic resolution of faba bean QTL intervals for ascochyta and orobanche resistance, particularly in chrs. II, III, V and VI. To this aim, a set of WRKY TFs and defense related proteins falling within the targeted QTL intervals were assayed with the aim of further refine the QTL regions and identify causal genes responsible for trait expression. To increase the rate of polymorphism in the target regions, a second RIL population segregating for resistance against both pathogens and sharing the male parental line (Vf6 x Vf136) was included in the analysis. Compilation of different favourable alleles of QTLs obtained from the different genetic backgrounds may help to develop effective strategies for the application of MAS. The fine-mapping approach proposed here increases the genetic resolution of relevant QTLs and paves the way for an efficient deployment of useful alleles for ascochyta and broomrape resistance through marker-assisted breeding.

## Results

### Genotyping of WRKY TFs and other defense related genes

Using candidate genes from syntenic regions of *Medicago*, we performed BLAST searches against the in-house faba bean transcriptome to retrieve orthologous faba bean candidates falling within or close to previously reported QTL regions controlling resistance to ascochyta and broomrape. The approach identified 219 positional candidate genes for plant responses to pathogens including WRKY transcription factors (30), NBS-LRR and receptor-like protein kinases (21), zinc finger proteins (6) as well as other candidate defense-related genes (Additional file 1). Genotyping of these candidate genes was performed either by KASPar assays or by the commercially available Sequenom MassArray iPLEX SNP platform. The genotyping results and the chromosome location of the polymorphic genes in the two RIL populations are depicted in the Additional files 1 and 2.

### Linkage analysis

The genotypic data from the disease resistance candidate markers were combined with the molecular data set previously reported in the 29H x Vf136 and Vf6 x Vf136 RIL populations [16, 23]. Both maternal lines have partial resistance to ascochyta blight and susceptibility to broomrape while the common male parent, Vf136, is susceptible to ascochyta and partially resistant to broomrape.

From the 219 candidates identified, 90 markers (41%) were found to be monomorphic in the 29H x Vf136 RIL population and most of these (84 markers) were derived from the KASPar assays. Eleven markers with more than 25% of missing values (probably due to technical genotyping errors), and 23 markers showing distorted segregation were discarded for further analysis, thus reducing the markers left for linkage mapping to 95 (Additional file 1). These 95 new markers were added to the previous data set [16] and 70 of them saturated the stable QTL regions in chrs. II, III, V and VI. The 25 remaining markers were distributed on the remaining chromosomes. Detailed information on the marker distribution and location per chromosome is provided in Table 1. The new 29H x Vf136 map contains 366 markers that span 3,387 cM in total with a mean distance of 9.25 cM per marker locus. A significant reduction in marker density was achieved in chrs. III and VI after saturation, where the average distance between adjacent markers now ranges between 4.5 and 5.4 cM respectively, compared with an average intermaker distance of 8 cM (Table 1).

**Table 1:** Information of the new map developed in the 29H x Vf136 population

Chromosome	Length (cM)	New markers mapped	Total n°. of markers mapped	Average marker density (cM)
**II**	**337.44**	**12**	**40**	**8,44**
**III**	**158.27**	**19**	**35**	**4,52**
**V**	**563.33**	**18**	**40**	**13,03**
**VI**	**266.79**	**21**	**49**	**5,41**
**Total**	**1,325.82**	**70**	**164**	**8,08**
I	203.41	3	18	11,3
I	37.18	1	6	6,2
I	64.11	1	9	7,12
I	374.02	0	32	11,69
I	80.29	0	11	7,3
I	195.95	1	14	14
IB	35.19	0	6	5,86
II	8.26	0	2	4,13
IIA	152.56	3	11	13,87
III	61.35	1	6	10,23
III	114.85	0	12	9,57
III	143.61	0	13	11,05
III	63.78	0	7	9,11
IV	88.59	4	11	8,05
IV	50.67	1	3	16,89
VI	51.06	2	4	12,76
VI	33.99	2	4	8,5
VI	5.07	0	2	2,54
VI	42.94	1	4	10,73
VI	17.96	0	3	5,99
VI	48.72	1	4	12,18
-	30.97	2	4	7,74
-	110.62	2	9	12,29
-	46.32	0	3	15,44
-	43.35	0	4	10,84
**Total**	**3,386.57**	**95**	**366**	**9,25**

The QTLs regions subjected to saturation in chromosomes II, III, V and VI, are higlighted in bold

In the Vf6 x Vf136 RIL population, 56 of the markers assayed could be genotyped and mapped in the progeny (Additional file 2). Fifty of them saturated the previous map [23], falling within the regions bearing stable QTLs in the target chromosomes. The new map with 332 markers covers 3,623 cM with a mean marker interval of 11 cM. After saturation, the average marker density is slightly lower only in chrs. II and III (10.29 and 9.16 cM, respectively) (Table 2).

**Table 2:** Information of the new map developed in the Vf6 x Vf136 population

Chromosome	Length (cM)	New markers mapped	Total no. of markers mapped	Average marker density (cM)
**II**	**731.29**	**15**	**71**	**10.29**
**III**	**577.29**	**14**	**63**	**9.16**
**V**	**357.69**	**8**	**31**	**11.53**
**VI**	**703.38**	**13**	**62**	**11.34**
**Total**	**2,369.65**	**50**	**227**	**10.58**
I	789.10	5	67	11.77
I	463.89	1	38	12.21
**Total**	**3,622.65**	**56**	**332**	**11.05**

The QTLs regions subjected to saturation in chromosomes II, III, V and VI are higlighted in bold

**Fig. 1:**
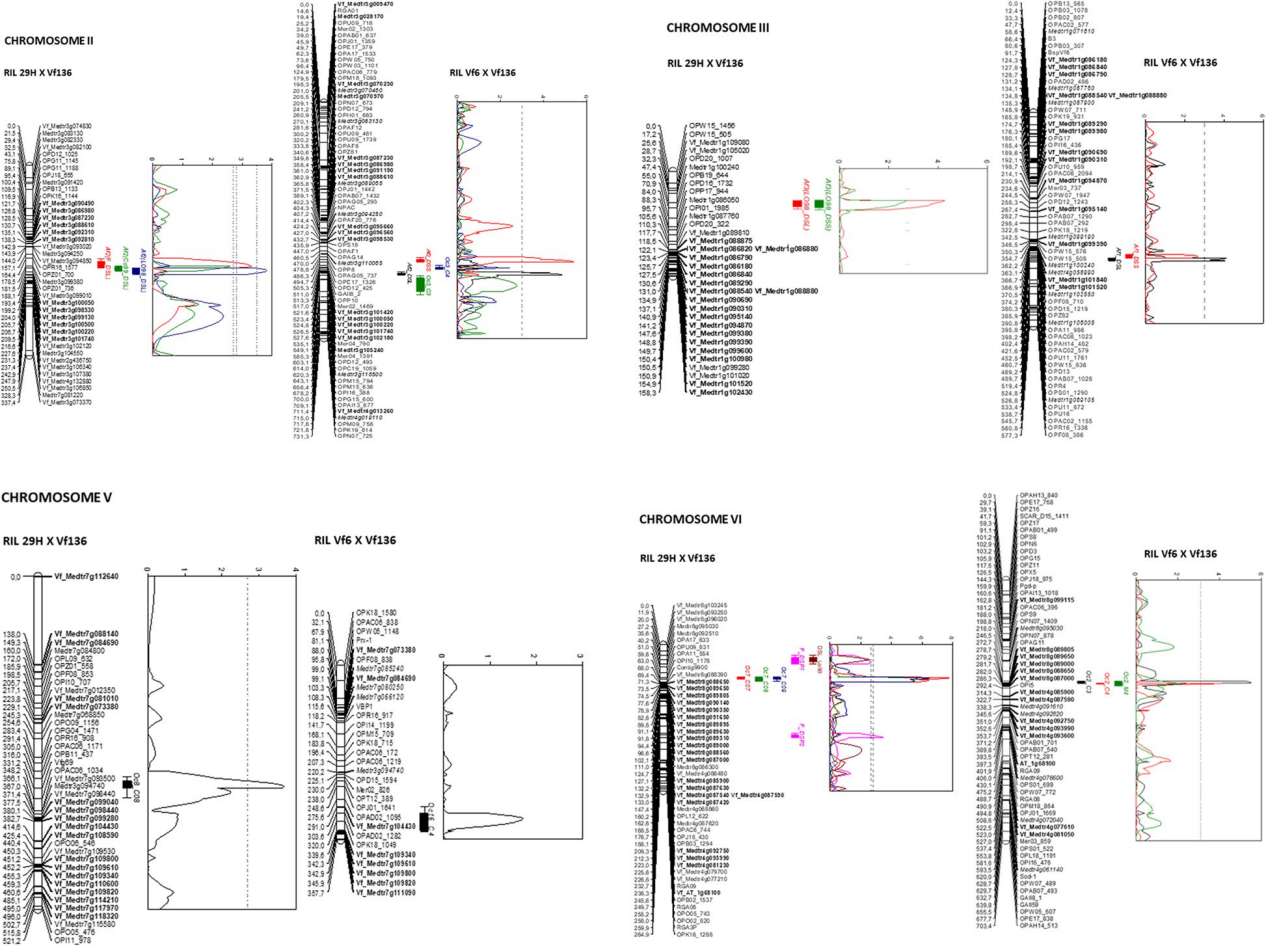
Linkage groups and QTLs detected in chrs. II, III, IV and VI from two faba bean RIL populations. 29H x Vf136 RIL population (left) and Vf6 x Vf136 RIL population (right). The positions of the new candidate genes are highlighted in bold and the markers renamed in the Vf6 x Vf136 population are in italics. Marker names are on the right and the estimated map distances are shown on the left. Recombinant fractions were converted to centiMorgans using the mapping function of Kosambi (1944)

The two linkage maps rebuilt in this paper contain a mixture of marker types, including RAPDs, SSRs, RGAs, EST-derived markers and the new defense related genes. To unify the marker nomenclature, the IDs of 34 markers used in the former Vf6 x Vf136 map [23] were renamed with the corresponding EPPO code (Medtr) for *Medicago* (Additional file 3). Both maps are in general collinear and share 40 common loci, which provide anchors to facilitate comparison of the QTLs mapped in the two populations. Figure 1 shows the linkage groups and QTLs detected in chrs. II, III, V and VI after saturation. The positions of the new candidate genes are highlighted in bold and the markers renamed in the Vf6 x Vf136 population are in italics.

### QTL analysis in the 29H x Vf136 RIL population

As expected, the MQM analysis in cross 29H x Vf136 revealed three QTL regions associated with ascochyta resistance in chrs. II (*Af*2), III (*Af*3) and VI. All the resistance-enhancing alleles originated from the resistant parent 29H as shown by the negative values of the additive genetic effects (Table 3).

**Table 3:** Results of MQM analysis for ascochyta and broomrape resistance in the RIL 29H x Vf136

QTLs	Enviroment	Chr	QTL interval	Interval length (cM)	Nearest locus to the max. LOD peak	LOD Threshold	Max. LOD score	% variation (R2)	Additive
Af2 (F_DSL)	Cordoba2005/06	II	OPZ01_700/OPZ01_736	17.1	Medtr3g099380	3.5	3.31	13.5	-4.48
Af2 (DSL_C99)	Cordoba1999/00	II	OPZ01_700/Vf_Medtr3g099010	23.7	Medtr3g099380	2.7	2.59	11.1	-1.78
Af2 (DSL_Lo98)	Logroño1998/99	II	Medtr3g099380/**Vf_Medtr3g100050**	14.88	Vf_Medtr3g099010	2.8	3.82	14.9	-1.92
Af3 (DSL_Lo98)	Logroño1998/99	III	**Vf_Medtr1g088875**/Medtr1g086050	30.11	OPD20_322	2.8	4.27	16	-1.96
Af3 (DSS_Lo98)	Logroño1998/99	III	**Vf_Medtr1g088875**/Medtr1g086050	30.11	OPD20_322	2.7	2.72	11.3	-0.91
Oc8_C8	Cordoba2007/08	V	OPB11_437/OPAC06_1034	32.17	Vfg69	2.7	3.68	14	-0.05
F_DSP1	Cordoba2005/06	VI	Vf_Medtr8g096020/Medtr8g092510	15.56	Medtr8g095030	2.8	2.7	11.7	-0.37
F_DSP2	Cordoba2005/06	VI	OPL12_622/OPJ18_430	16.47	Medtr4g087620	2.8	3.5	14	-0.39
DSL_Lo98	Logroño1998/99	VI	Vf_Medtr8g096020/Medtr8g092510	15.56	Medtr8g095030	2.8	2.61	7.8	-1.43
Oc7_C07	Cordoba2006/07	VI	OPU09_831/OPI10_1178	33.82	OPA11_554	2.8	7.76	33	-0.09
Oc7_C08	Cordoba2007/08	VI	OPU09_831/Vf_Medtr8g088390	18.38	OPA11_554	2.7	5.72	23.3	-0.07
Oc7_C09	Cordoba2008/09	VI	OPU09_831/Vf_Medtr8g088390	18.38	OPA11_554	2.8	6.49	27.1	-0.08

The markers mapped in this study are higlighted in bold

In chr. II, QTL *Af*2 scored as the ascochyta disease severity in leaves (DSL), was consistently detected in the three years of evaluation under both conditions tested (growth chamber and field trials). F_DSL, DSL_C99 and DSL_Lo98 explained between 11.1% and 14.9% of the total phenotypic variation (Fig. 1; Table 3). Chr. II was enriched with 12 candidate genes and, considering the *M. truncatula* genetic distances, the *Af*2 QTL interval was strongly reduced from 6.58 Mb encompassing 748 putative candidate genes [16] to 1.68 Mb and 191 genes (Table 4). The closest loci to the maximum LOD peaks are Medtr3g099380 (annotated as a 14-3-3 like protein) and Vf_Medtr3g099010 (a HVA22-like protein). 14-3-3 proteins play central roles in the regulation of signal transduction and diverse cellular processes, ranging from metabolism to transport, growth, development and stress response. In plants, they are required for disease resistance and represent key targets of pathogen effectors. For example, several 14-3-3 isoforms in tomato contribute to Xv3 disease resistance to the bacterial pathogen *Xanthomonas euvesicatori*a [24], while in rice several 14-3-3/GF14 proteins were differentially regulated during the interactions with *Xanthomonas oryzae* and the fungal pathogen *Magnaporthe oryzae* [25].

**Table 4:** Ascochyta and broomrape resistance QTL intervals in cross 29H x Vf136 achieved in this study

Trait	Vicia faba chromosome	QTL interval (Ocaña-Moral et al. 2017)	Distance (Mb) in Medicago Truncatula	No. genes in Medicago truncatula	QTL interval in present study	Distance (Mb) in Medicago Truncatula	No. genes in Medicago truncatula
Af2 (DSL)	II	Medtr3g091420 - Medtr3g104550	6.58 Mb	881	Medtr3g094850 - Medtr3g098530	1.68 Mb	226
Af3 (DSL /DSS)	III	Medtr1g086050 - Medtr1g100240	6.51 Mb	840	Medtr1g086050 - Medtr1g088875	1.35 Mb	182
Oc8	V	Medtr7g098250 - Medtr7g118320	9.78 Mb	1.287	Medtr7g073380 - Medtr7g093500	9.92 Mb	1.233
DSL/DSP1	VI	Medtr8g092510 - Medtr8g103245	4.78 Mb	653	Medtr8g092510 - Medtr8g096020	1.35 Mb	182
Oc7	VI	Medtr8g088390 - Medtr8g092510	2.14 Mb	312	Medtr8g088650 - Medtr8g092510	1.94 Mb	283
DSP2	VI	Medtr4g068860 - Medtr4g091610	10.25 Mb	1.396	Medtr4g087420 - Medtr4g092750	2.43 Mb	345

Genomic distance and estimated number of genes involved as compares with previously reported QTL interval data (Ocaña et al. 2017)

In chr. III, only two of the six previously reported QTLs, DSL_Lo98 and DSS_Lo98 [10, 26], were significant explaining 16 and 11.3% of the phenotypic variation, respectively (Fig. 1; Table 3). Despite the saturation of the target region with 19 candidates and the concomitant reduction of the QTL interval from 6.5 to 1.34 Mb and number of candidate genes from 736 to 148 (Table 4), a RAPD marker (OPD20_322) remains the closest known locus to the *Af*3 LOD peak.

Finally, in chr. VI three QTLs (F_DSP1, F_DSP2 and DSL_Lo98) were detected, in total agreement with the previous study [16]. The percentage of variation explained by these QTLs ranged from 7.8% to 14%. This was the most highly saturated QTL region with 21 new candidates. Accordingly, the QTL interval and number of possible candidates were reduced from 10 Mb and 1165 genes to 2.43 Mb and 270 genes (Table 4). The closest loci to the maximum LOD peaks were Medtr8g095030 and Medtr4g087620 (Table 3), both of which represent promising candidates for further research. Medtr8g095030 encodes a leucine-rich repeat receptor-like serine/threonine-protein kinase (LRR-RLK), which are part of the pattern recognition receptors (PRRs) on the plant cell surface that recognize pathogen-associated molecular patterns (PAMPs) to elicit the first-layer immune response [27, 28]. LRR-RLKs protein kinases regulate plant growth and development, hormone signal transduction and response to biotic or abiotic stresses [29]. On the other hand, Medtr4g087620 is annotated as a homolog of the mitogen-activated protein kinase (MAPK) MMK1. MAPKs are conserved signal transduction components in fungi, plants, and mammals. During the interaction between phytopathogenic fungi and plants, fungal MAPKs help to promote mechanical and/or enzymatic penetration of host tissues, while plant MAPKs are required for activation of plant immunity. Plant MAPKs promote defense mechanisms that threaten the survival of fungal cells, leading to a stress response mediated in part by fungal MAPK cascades [30]. While early studies focused mainly on MAPKs in model plants, whole-genome sequencing studies revealed that MAPK cascades play crucial roles in biotic and abiotic stress responses of different crops [31].

Concerning resistance to *O. crenata,* in line with the previous study [16] the MQM analysis only detected two QTLs, *Oc*7 and *Oc*8. The most relevant QTL (*Oc*7) in chr. VI was detected during the three years of evaluation explaining between 23.3% and 33% of the phenotypic variation (Table 3; Fig. 1). The LOD scores ranged from 5.72 to 7.76, largely exceeding the LOD significance threshold of 2.8 and were the highest detected in this study. As mentioned above, despite saturating the QTL interval with 12 candidates, neither the genetic distance nor the estimated number of genes encompassed by the QTL interval were significantly reduced (Table 4). Nevertheless, one of the former flanking RAPD markers was replaced by Vf_Medtr8g088390, encoding a *Medicago* YTH domain-containing protein. YTH domain proteins play important roles in multiple aspects of plant life such as development and responses to abiotic and biotic stress [32]. Overexpression of YTH proteins MhYTP1 or MhYTP2 in *Malus hupehensis* led to increased sensitivity to heat stress, high salinity and pathogen infections. However, no biological functions of YTH domain-containing proteins have been reported in other plant species [33]. The second QTL for *O. crenata* resistance, *Oc*8 in chr. V, was only detected in one season (2007-08) accounting for 14% of the variation (Table 3). As for *Oc*7, the saturation of the *Oc*8 target region with 18 new defense related genes did not significantly affect the position of the QTL and the closest locus to the maximum LOD peak, the microsatellite Vfg69 derived from the Orobanche resistant cultivar Giza 402 [34].

### QTL analysis in the Vf6x Vf136 RIL population

To increase the level of polymorphism and to compare the position of relevant QTLs for ascochyta and broomrape resistance in different genetic backgrounds, the candidate genes identified in the 29H x Vf136 population were subsequently tested for polymorphisms and QTL mapping in cross Vf6 x Vf136. Chrs. II, III, V and VI were saturated with 50 new markers, most of which localized in close vicinity of the targeted QTL regions to replace previous, uninformative RAPD markers with consistent and reliable SNPs in candidate genes (Fig. 1; Table 2). The approach allowed for the first time to define the QTL intervals with gene derived markers. The genomic distance of each interval and the number of genes included are shown in Table 5.

**Table 5:** Ascochyta and broomrape resistance QTL intervals in cross Vf6 x Vf136 reported for the first time in this study

Trait	Vicia faba chromosome	QTL interval in present study	Distance (Mb) in Medicago Truncatula	No. genes in Medicago truncatula
Af2 (DSS/DSL)	II	Medtr3g105240-Medtr3g110065	2.37 Mb	334
Oc3	II	Vf_Medtr3g102180-Medtr3g116500	7.36 Mb	989
Af1 (DSS/DSL)	III	Medtr1g102550-Medtr1g106005	1.63 Mb	237
Oc16	V	Vf_Medtr7g104430-Vf_Medtr7g109340	2.36 Mb	330
Oc2	VI	Vf_Medtr8g087000-Medtr8g095030	3.70 Mb	525
Oc5	VI	Vf_Medtr4g085900-Vf_Medtr4g091610	2.69 Mb	370

In agreement with previous study [12], two QTLs in chr. II (*Af*2) and III (*Af*1) were detected for ascochyta resistance both in leaves (DSL) and stems (DSS) (Table 6). *Af*2 QTLs explained around 15% of the phenotypic variation and the closest locus to the LOD peak in *Af*2_DSL is now Vf_Medtr3g102180, a predicted inactive receptor kinase which appears to be a promising candidate. Receptor kinases play crucial roles in plant immunity, growth and development and are involved in responses to different stress conditions such as mechanical wounding and pathogen infection as part of the first layer of inducible defense [35]. In addition to *Af*2, two other QTLs for *O. crenata* resistance (*Oc*3_C3 and *Oc*3_C4) were detected in chr. II but their respective LOD scores (2.76 and 1.7) did not reach statistical significance (Table 6). However, the LOD value of *Oc*3_C3 was above the threshold of 2 fixed for significance in the previous study [23].

**Table 6:** Results of MQM analysis for ascochyta and broomrape resistance in the RIL Vf6 x Vf136

QTLs	Enviroment	Chr	QTL interval	Interval length (cM)	Nearest locus to the max. LOD peak	LOD Threshold	Max. LOD score	% variation (R2)	Additive
Af2_DSL	Cordoba2002/03	II	**Vf_Medtr3g101740**/Mer04_790	9.3	**Vf_Medtr3g102180**	3	4.86	14.9	-1.28
Af2_DSS		II	OPAG05_737/OPE17_1326	15.92	OPE17_1326	3.1	5.41	15.8	-4.02
Oc3_C3	Cordoba2002/03	II	Mer04_790/Mer04_1391	50.18	Medtr1g071610	3.1	2.76	13.4	-0.07
Oc3_C4	Cordoba2003/04	II	OPD12_493/Medtr3g116500	17.18	OPC19_1059	3.1	1.7	4.4	-0.04
Af1_DSL	Cordoba2002/03	III	OPZ82/OPAC06_1023	12.31	Medtr1g106005	3	4.12	12.1	-1.17
Af1_DSS		III	OPD15_1219/Medtr1g106005	10.63	OPZ82	3.1	2.67	7.8	-2.82
Oc16_C4	Cordoba2003/04	V	OPAD02_1282/Vf_Medtr7g109340	35.99	OPK18_1049	3.1	1.71	5.3	-0.05
Oc2_C3	Cordoba2002/03	VI	OPAG11/**Vf_Medtr8g089000**	9.18	**Vf_Medtr8g089805**	3.1	5.5	16.6	-0.08
Oc2_C4	Cordoba2003/04	VI	OPAG11/**Vf_Medtr8g089000**	7.54	**Vf_Medtr8g089805**	3.1	3.98	13	-0.07
Oc2_M4	Mengibar2003/04	VI	OPAG11/**Vf_Medtr8g087000**	13.58	**Vf_Medtr8g089650**	3	2.1	6.4	-0.05

The markers mapped in this study are higlighted in bold

In chr. III, only QTLs for ascochyta resistance in leaves and stems were identified. *Af*1_DSL was significant accounting for 12.1% of the phenotypic variation, while *Af*1_DSS, the second QTL previously reported in this region, did not reach statistical significance in this study. Both QTLs share a common flanking marker (Medtr1g106005) which is annotated as an α-tubulin that together with β-tubulin forms protofilaments, the main component of the microtubule cytoskeleton. A growing number of studies suggest that in addition to playing vital roles in mechanical architecture and cell division, microtubules can act as sensors for stress response signaling in plants. Thus, in *Arabidopsis* microtubule depolymerization and reorganization was shown to be essential for enhancing plant tolerance to salt stress [36]. However, the mechanisms that integrate microtubule regulation, cellular metabolism and stress response signaling remain unclear [37].

As was previously reported [38], a single QTL for *O. crenata* resistance (*Oc*16_C4) in the distal part of chr. V explained 5.3% of the phenotypic variation although it did not not reach the defined threshold (Table 6). Nevertheless, the location of *Oc*16_C4 in close vicinity with syntenic markers in the *Oc*8 interval in cross 29H x Vf136 (Fig. 1), reinforces the homology of both QTLs and their possible role in plant defence. After QTL saturation, a previous RAPD flanking *Oc*16_C4 was replaced by Vf_Medtr7g109340, encoding a LRR RLK. Nevertheless, since the closest QTL peak locus remains a RAPD (OPK18_1049) we cannot rule out the possibility that other candidate genes underlie this QTL.

Finally, QTL *Oc*2 in chr. VI, which was previously reported to confer broomrape resistance [23, 38], was significant in two (*Oc*2_C3 and *Oc*2_C4) of the three field trials assayed accounting for 16.6% and 13% of the phenotypic variation, respectively (Table 6). After saturating the region, the closest RAPD marker to the LOD peak was replaced by Vf_Medtr8g089805 while in case of the third QTL (*Oc*2_M4), it was replaced by Vf_Medtr8g089650. *Oc*2_M4 did not reach the significance level, but the precise colocalization of the three QTLs highlight the interest of this genomic region for further fine mapping, candidate gene identification and marker-assisted breeding. Vf_Medtr8g089805 is annotated as a GDSL esterase/lipase. Accumulating evidence suggests lipids as important regulators of plant defense [39, 40] and several reviews report the antimicrobial activity of GDSL lipase/esterases in local and systemic acquired resistance (SAR) [41–43]. Among multiple examples, two rice GDSL lipases that modulate immunity through lipid homeostasis were identified [44]. GDSL lipases were shown to possess anti-microbial activity and to modulate resistance to *Alternaria* in association with ethylene signaling in Arabidopsis [45] and identified as potential candidates for improving salinity and drought tolerance in soybean [46]. The remaining QTL peak marker (Vf_Medtr8g089650) encodes a predicted member of the WD40 domain proteins, which participate in a wide range of cellular processes such as signal transduction, transcriptional regulation, plant organ development and defense responses [47]. Thus, both QTL peak markers point towards promising candidates mediating resistance against Orobanche.

## Discussion

Several studies have identified key players in faba bean resistance to *a*scochyta and broomrape and validated significant QTLs in two RIL populations 29H x Vf136 and Vf6 x Vf136 [8–13]. Nevertheless, the giant faba bean size (13 Gb) and the traditional less informative markers (RAPDs, SSRs) used in earlier map versions make it difficult to pinpoint causative loci responsible for specific traits. Subsequent efforts using innovative strategies such as synteny, translational genomics or transcriptome sequencing resulted in enhanced genetic resolution of the maps [14–16, 38], although the QTL intervals were still too large to reduce the number of positional candidate genes for further functional analysis.

Marker saturation of chromosomal regions affecting a specific trait is one of the first steps for improving the precision for marker-assisted selection and QTL pyramiding. Here we extended the QTL mapping and saturation of target genomic regions controlling ascochyta and broomrape resistance in two genetic backgrounds by selecting 219 WRKYs, TFs and defense related proteins from *Medicago* that fell within or close to previously reported QTL intervals with the aim of identifying and mapping faba bean orthologous in comparative genomic regions. The preliminary 29H x Vf136 map [16] included 257 loci spanning 2,796.91 cM, with the QTLs for *Oc* and *Af* mostly flanked by anonymous RAPD markers. In this study, the inclusion of 95 new gene markers allowed the construction of a more saturated genetic map (3,386.57 cM) and improved the coverage particularly in chrs. II, III and VI. As a result, the QTL intervals in these chromosomes were narrowed by 5x (ranging from 1.35 Mb in chr. III to 2.43 Mb in chr. VI) and the number of possible candidate genes was reduced accordingly (Table 4). Our approach identified several QTL peak markers that are significantly associated with ascochyta and broomrape resistance. Thus, in chr. II. and VI, the closest loci to the maximum LOD peaks in *Af* QTLs were Medtr3g099380 (annotated as a 14-3-3 like protein), Vf_Medtr3g099010 (HVA22-like protein), Medtr8g095030 (LRR RLK) and Medtr4g087620 (MAPK), all of which represent promising candidates for further research.

A similar outcome was obtained in the enriched Vf6 x Vf136 map. While the previous map developed [23] included 277 mostly uninformative RAPD markers that covered 2,856.7 cM, saturation with 56 candidates resulted in a total length of 3,622.64 cM. Apart from saturating QTL target regions, the approach allowed for the first time to define QTL intervals based on orthologous and collinear *Medicago* defence-related genes (Table 5). Except for *Oc3* in chr. II, the remaining QTL intervals range now from 1.63 Mb (*Af*1_DSS/DSL in chr. III) to 3.7 Mb (*Oc*2 in chr. VI) (Table 5). The closest locus to the LOD peak for *Af*2_DSL in chr. II is Vf_Medtr3g102180, a predicted inactive receptor kinase that was shown to play crucial roles in plant immunity, growth and development. In chr. III the *Af1* QTLs share a common marker (Medtr1g106005) annotated as an α-tubulin with possible role in microtubule regulation, cellular metabolism and plant stress response signaling. Finally, in chr. VI the significant *Oc*2 QTLs expose a relevant genomic region homologous to *Oc*7 in cross 29H x Vf136 for further fine mapping. One of the LOD peak markers (Vf_Medtr8g089805) is a GDSL esterase/lipase with a reported antimicrobial activity in local and systemic acquired resistance (SAR), while Vf_Medtr8g089650 is a transducin/WD40 repeat protein regarded as crucial regulator of plant developmental processes (Table 6).

Identification of common QTL across different studies (eg. *Oc*2 = *Oc*7; *Oc*8 = *Oc*16; *Af*1 = *Af*3 and *Af*2) is of high interest since these QTL are most likely to be robust across different environments and pedigrees and should thus be highly amenable to improvement through plant breeding and marker-assisted selection [48]. Although they may not be the causal genes, the candidates mapped here, including those that collocate with the QTL peaks, provide additional informative markers and will facilitate identification of new candidates in relevant target regions.

## Conclusions

The current study extends our previous work [9, 10, 12, 16, 23] by including defence-related candidate gene loci to QTL regions relevant for ascochyta and broomrape resistance. The synteny approach with *Medicago* successfully identified promising positional candidates associated with resistance to both pathogens. The addition of new markers allowed us to: (i) replace previous dominant and pedigree-specific RAPD markers by more robust and transferrable SNP markers, (ii) delimit the QTL regions to smaller intervals compared with previously published data and (iii) define closer flanking markers for further fine mapping. Markers linked to the QTLs revealed significant allelic and genotypic differences suggesting that they are potentially useful in MAS, at least in the genetic backgrounds described. Further research will include fine mapping, association mapping and expression studies, which together with the upcoming faba bean reference genome will greatly help to identify the genes controlling *Ascochyta fabae* and broomrape resistance in this crop.

## Methods

### Mapping populations

The most recent maps of two contrasted RIL populations, 29H × Vf136 [16] and Vf6 × Vf136 [23] segregating for resistance to both broomrape and *A. fabae* were used in the study. The populations consisted of 119 and 165 F_7:8_ individuals, respectively, developed by single seed descendents in insect proof cages. The genotype 29H is a minor type from INRA (France) originally provided by Drs. Berthelem and Le Guen (Station d’Amélioration des Plantes, INRA-Rennes, France). This genotype was described as resistant to *A. fabae* [49–52] and susceptible to broomrape [9]. The male parent, Vf136 is an equina type obtained at IFAPA (Córdoba) from the cross Vf1071 × Alameda [53]. This genotype is susceptible to *A. fabae* and resistant to broomrape [9, 12, 23, 26, 54]. The other female parent Vf6 is an equina asynaptic line belonging to IFAPA, resistant to ascochyta and susceptible to the parasite. Seeds from these materials are conserved at the IFAPA germplasm bank.

### Marker selection and genotyping

BLAST searches were performed to mine the WRKY genes, resistance gene analogs (RGAs) and resistance protein sequences annotated in the faba bean in-house transcriptome [15]. In addition, we exploited the synteny with *M. truncatula* (www.medicagohapmap.org/fgb2/gbrowse/mt40/) for further saturation of the target regions with other gene categories and transcription factors reported to be involved in plant resistance to pathogens (eg. phytohormones, pathogenesis related (PR) genes, classical defense-response genes involved in cell-wall fortification and the phenylpopanoid pathway, defensins, etc.). Based on the physical positions of single nucleotide polymorphism (SNP) markers and gene annotation in *M. truncatula*, 219 candidate gene sequences falling within or close to the putative QTL syntenic regions in the previous 29H x Vf136 map [16] were identified. The interval ranges considered, the genomic distance and the estimated number of *Medicago* genes included in these intervals are presented in Table 4. Gene sequences were aligned to identify single nucleotide polymorphisms (SNPs) between the parental lines and further genotyped in the RIL population. Detailed information of the selected genes is shown in Additional file 1.

Finally, to increase the rate of polymorphism and to compare relevant QTLs regions between different genetic backgrounds, the positional pathogenesis related candidates in the RIL 29H x Vf136 were further genotyped and mapped in a second faba bean RIL population (Vf6 x Vf136) segregating as well for both disease resistance traits (Additional file 2).

SNP genotyping was performed using Kompetitive Allele Specific PCR (KASPar) assays [55] provided by LGC genomics (www.lgcgenomics.com/genotyping/kasp-genotyping-chemistry) and the MassArray iPLEX (Sequenom) SNP typing platform at the Spanish National Genotyping Center facility of the University of Santiago de Compostela (www.usc.es/cegen/). KASP is a fluorescence-based genotyping technology based on allele-specific oligo-extension and fluorescence resonance energy transfer for signal generation [56]. MassArray iPLEX consists of an initial locus-specific polymerase chain reaction (PCR), followed by single-base extension using mass-modified dideoxynucleotide terminators of an oligonucleotide primer that anneals immediately upstream of the polymorphic site of interest [57].

### Linkage map construction and QTL analysis

Polymorphic markers genotyped in both populations were incorporated into previous marker datasets [16, 23] to develop new linkage maps. Segregating data were analyzed for goodness of fit to the expected 1:1 ratio using the chi-square test and the JoinMap version 4.0 software was used for linkage map construction [58, 59]. Markers were grouped using the maximum likelihood option at a minimum LOD score of 4.0 and maximum recombination fraction of 0.25. Recombination fractions were converted to centimorgans (cM) using the mapping function of [58, 59].

QTL analysis was conducted using the MAPQTL 5 software [60]. Considering previous evaluation performed in both faba bean populations, QTLs for disease resistance were identified using interval mapping (IM). An initial set of cofactors was selected from the interval mapping results, and a backward elimination procedure was used to select significant markers. Only markers significant at *P* = 0.01 were used as cofactors in the multiple QTL analysis (MQM) [61]. The threshold for the detection of a QTL was determined using 1000 permutations [62] and a significance (*P*-value) level of 0.05. The presence of a QTL was accepted based on its P-value and co-location with QTLs for different resistance traits. An estimation of the additive effect and the total variance explained (R2) by the QTL at the position with the highest LOD score was given by MapQTL 5.0. Uncertainty of the QTL position was indicated by a 1-LOD and 2-LOD support intervals [63–65]. MapChart software [65] was used to produce the QTLs figures for ascochyta and broomrape resistance.

### Defining the QTL intervals

According to the results of the new QTL analysis, we redefined the QTLs interval based on the nearest *Medicago* genes flanking the QTL positions. To know the function and the physical position of each gene we use the Gene database (www.ncbi.nlm.nih.gov/gene/). The physical location of these markers was used to determine the genomic distance spanned (Mb) for each QTL and the number of potential candidate genes encompassed by each interval using Genome Data Viewer (GDV) (www.ncbi.nlm.nih.gov/genome/gdv/).

### Field trials and resistance scoring

In the RIL population 29Hx Vf136, we used the ascochyta and *Orobanche crenata* resistance scores previously reported [9, 10]. *Ascochyta blight* resistance was determined in three different experiments described in previous works [10, 26]. The first two tests were carried out at seedling stage in growth chambers and against two monocodial isolates, C99 and Lo98, collected in Córdoba and Logroño (Spain), respectively. Fifteen days after inoculation, disease scoring was performed separately on leaves (DSL, disease severity on leaves) and stems (DSS, disease severity on stems) based on the percentage of symptomatic leaf area. The third disease test was performed in adult plants grown in field trials (F) at Córdoba, Spain, in 2005-06 using a local isolate. The disease infection was determined on leaves (DSL), stems (DSS) and pods (DSP). Furthermore, broomrape resistance was evaluated in naturally infested fields in Córdoba (Spain) in three consecutive seasons (2006-2009). Field trial design and resistance scoring were fully described in previous study [9]. In brief, resistance to broomrape was scored as the final number of emerged broomrapes per faba bean plant grown in a randomized design with two replications, all including a susceptible check.

In cross Vf6 x Vf136, phenotype data for ascochyta and broomrape QTL analysis was selected from published works of the group [12, 13]. The RIL population was checked for ascochyta blight resistance in growth-chamber conditions described in previous work [12], using the monoconidial isolate CO95–01 from Córdoba (Spain). Besides, the *O. crenata* evaluations were performed in naturally infested fields in two locations in Southern Spain (Córdoba and Mengibar) and two consecutive seasons 2002–2003 and 2003–2004. Evaluation methods and abbreviations were assigned according to the previous references.

## Supplementary Information

**Additional file 1.** List of candidate genes assayed to saturate the QTLs regions in the 29H x 136 RIL population detailing the genotyping method used, ID in the faba bean transcriptome (Ocaña et al. 2015), homologous gene in Medicago truncatula, protein function, chromosome location and genotyping results in this population.

**Additional file 2.** List of candidate genes assayed to saturate QTLs regions in the Vf6 x 136 RIL population detailing the genotyping method used, ID in the faba bean transcriptome (Ocaña et al. 2015), homologous gene in Medicago truncatula, protein function, chromosome location, genotyping results in cross 29H x 136 and common markers between the two RILs populations.

**Additional file 3.** Corresponding Medicago truncatula EPPO code (Medtr) of the 34 markers used in the former Vf6 x Vf136 map (Díaz-Ruiz et al. 2010).

## Data Availability

Data generated or analysed during this study are included in this published article and its supplementary information files. Raw data used in the analysis are available under request.

## Abbreviations

QTL: Quantitative Trait Loci
RIL: Recombinant Inbred Line
TF: Transcription Factor
RAPD: Rapid Amplified Polymorphic DNA
SNP: Single Nucleotide Polymorphism
MAS: Marker assisted selection
Gb: Gigabyte
JA: Jasmonic Acid
LRR: Leucine-Rich Repeat
RGA2: Rho2 GTPase-activating protein 2
Chr: Chromosome
cM: Centimorgan
SA: Salicylic Acid
ET: Ethylene
CaWRKYs: *Cicer arietinum* WRKYs
BLAST: Basic Local Alignment Search Tool
NBS: Nucleotide-Binding Site
KASPar: Competitive Allele-Specific PCR amplification
SSR: Simple Sequence Repeat
RGA: Resistant Gene Analog
EST: Expressed Sequence Tag
ID: Identification
EPPO: European and Mediterranean Plant Protection Organization
Medtr: Medicago truncatula
MQM: Multiple QTL Mapping
DSL: Disease Severity on Leaves
DSS: Disease Severity on Stems
LOD: Logarithm of the odd
PRRs: Pattern Recognition Receptors
PAMPs: Pathogen-Associated Molecular Patterns
MAPK: Mitogen-activated protein kinase
SAR: Systemic acquired resistance
PR: Pathogenesis related
Mb: Megabase
GDV: Genome Data Viewer
